# Reactive lymphoid hyperplasia of the liver after surgery for advanced sigmoid colon cancer: a case report

**DOI:** 10.1093/jscr/rjae248

**Published:** 2024-04-24

**Authors:** Ken Sunamura, Yutaka Endo, Koki Hayashi, Yusuke Uchi, Soji Ozawa, Motohide Shimazu

**Affiliations:** Department of Surgery, Tama Kyuryo Hospital, Tokyo 194-0202, Japan; Department of Surgery, Tama Kyuryo Hospital, Tokyo 194-0202, Japan; Department of Surgery, Tama Kyuryo Hospital, Tokyo 194-0202, Japan; Department of Surgery, Tama Kyuryo Hospital, Tokyo 194-0202, Japan; Department of Surgery, Tama Kyuryo Hospital, Tokyo 194-0202, Japan; Department of Surgery, Tama Kyuryo Hospital, Tokyo 194-0202, Japan

**Keywords:** reactive lymphoid hyperplasia, colorectal liver metastases, liver resection

## Abstract

We report a case of reactive lymphoid hyperplasia (RLH) mimicking colorectal liver metastases (CRLM) on preoperative workup that was clinically indistinguishable. A 78-year-old woman was found to have locally-advanced sigmoid cancer (T4), and then treated with radical sigmoidectomy. One year after the surgery, plain computed tomography (CT) revealed a low-density area in the right hepatic lobe. Metastatic liver tumors could not be ruled out with CT/ magnetic resonant imaging (MRI) and positron emission tomography–CT . Based on these findings, the patient was diagnosed with CRLM at S7 of the liver. The patient underwent right posterior sectionectomy. The tumor was adjacent to the right hepatic vein; however, no invasion was observed. The patient was pathologically diagnosed as having RLH. The patient showed no signs of recurrence 16 months after initial surgery. RLH is clinically indistinguishable from CRLM. Further evaluation is required to elucidate the effective strategies of detecting and treating hepatic RLH.

## Introduction

Reactive lymphoid hyperplasia (RLH) is thought to represent an immune reaction. RLH has been reported to occur in the gastrointestinal tract or skin; however, it has rarely been reported in the liver [1, 2]. To the best of our knowledge, about 84 cases of RLH in the liver have been reported to date [3, 4]. The imaging characteristics of RLH and colorectal liver metastases (CRLM) share common features, such as low-density appearance on contrast-enhanced computed tomography (CT), hypointensity on T1-weighted magnetic resonant imaging (MRI), and hyperintensity on T2-weighted MRI [5]. As such, the preoperative diagnosis of RLH becomes more challenging in patients with a history of colorectal cancer, as RLH closely resembles CRLM on both CT and MRI. Despite this, only a limited number of reports have addressed these specific diagnostic challenges. Herein, we report a case of RLH mimicking CRLM on preoperative workup that was clinically indistinguishable from liver metastasis.

## Case report

**Figure 1:**
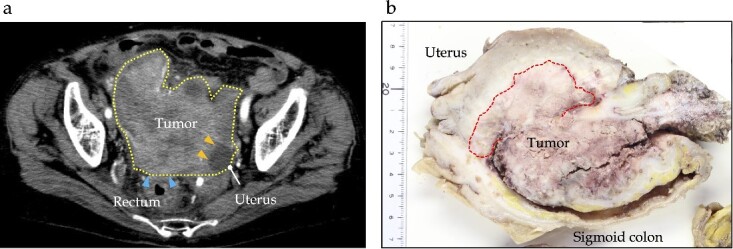
Preoperative and pathological findings of sigmoid carcinoma. (a) Contrast-enhanced computed tomography reveals a hypodensity mass (dotted line) of ~12 cm, located in the sigmoid colon, suspiciously invading the rectum (arrows) and uterus (yellow arrows). (b) Macroscopic findings reveal a type 1 tumor measuring 130 × 40 × 25 mm in the sigmoid colon, with direct invasion into the uterine corpus, left ovary, and small intestine.

A 78-year-old woman with no significant medical history was hospitalized with diarrhea. CT revealed a 13 cm tumor in the sigmoid colon with suspected invasion of the uterus and ovaries, which caused bowel obstruction (Fig. 1). After diverting transverse colostomy, the patient underwent a lower anterior resection with a combination of total hysterectomy, bilateral salpingo-oophorectomy, and partial enterectomy for a complete resection of tumor. The final pathology showed that the lesion was 130 × 40 × 25 mm in size, with a well-differentiated adenocarcinoma without lymph node metastases (T4bN0M0 and Stage IIC, based on the 8th Union for International Cancer Control staging). This patient received adjuvant chemotherapy for 6 months following the initial surgery [oral uracil-tegafur (UFT) with leucovorin (LV): UFT 400 mg/day and LV 75 mg/day on Days 1–28, every 35 days for five courses].

**Figure 2:**
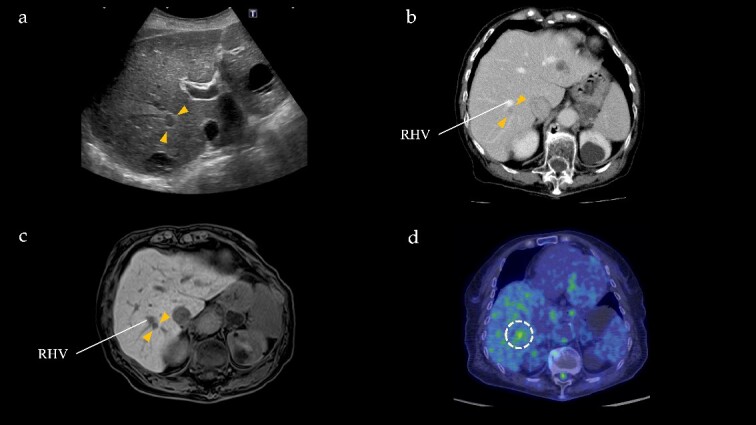
Radiographical findings of Segment 7 lesion in the liver. (a) On abdominal ultrasound, a hypoechoic tumor, measuring 10 mm (arrows), is identified in Segment 7. (b) Contrast-enhanced computed tomography indicates a 10 mm, hypodensity lesion located in Segment 7 (arrows) at portal phase. This lesion is close to the RHV. (c) Gadoxetic acid-enhanced magnetic resonance imaging shows a 10 mm, hypointensity lesion (arrows) in Segment 7 adjacent to the RHV at hepatic phase. (d) PET/CT reveals FDG accumulation (maximum standardized uptake value, SUVmax = 3.91) in the Segment 7 nodule.

**Figure 3:**
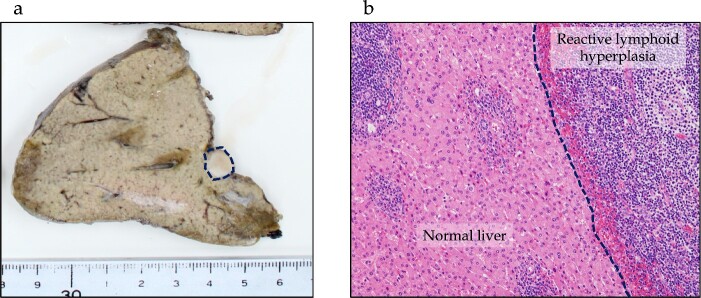
Pathological findings of RLH of the liver. (a) In the macroscopic findings, a whitish, 10 mm tumor is observed in Segment 7 of the liver (dotted line). (b) Microscopic findings show lymphocyte clusters with multiple germinal centers without any atypical lymphocytes, suggesting RLH. No residual lesions are seen on the resection margin.

One year after the initial surgery, a tumor in Segment 7 (S7) of the liver was detected on CT. The serum hepatitis B surface antigen and hepatitis C antibody test results were negative. Laboratory data on admission, including liver function test results, were unremarkable, including carcinoembryonic antigen (CEA) and CA19-9 levels. The imaging results are shown in Fig. 2. Abdominal ultrasonography revealed a hypoechoic, 10 mm diameter mass in the right hepatic lobe (S7). Plain CT revealed a low-density area in the right hepatic lobe (S7), presenting as a mass with reduced density in both the early and portal phases with contrast enhancement. Gadoxetic acid (Gd-EOB-DTPA)-enhanced MRI (EOB-MRI) revealed a slightly low-intensity lesion on T1-weighted images and a high-intensity lesion on T2-weighted images. Positron emission tomography–computed tomography (PET/CT) showed fluorodeoxyglucose (FDG) accumulation (maximum standardized uptake value, SUVmax = 3.91) in a nodule of the liver S7. We diagnosed this lesion as CRLM at S7. The patient underwent right posterior sectionectomy. The right posterior Glissonian pedicle was isolated extrahepatically. The tumor was adjacent to the right hepatic vein (RHV); however, no invasion was observed. The operative time was 302 min, and the estimated blood loss was 321 ml. Macroscopic examination revealed a white mass measuring 8 × 9 × 8 mm. Microscopic findings revealed clusters of lymphocytes with multiple germinal centers (shown in Fig. 3). There was no atypia in the lymphocytes, and aggregation was thought to be due to lymphocyte reactivity, consistent with RLH. There was a portal vein area at the edge of the tumor, and bile ducts were identified, but there were no findings suggestive of malignancy such as degeneration or irregular arrangement. According to these findings, this patient was diagnosed as RLH. The patient was discharged 11 days postoperatively with good progress. The patient showed no signs of recurrence at 40 months after the initial surgery.

## Discussion

We observed RLH of the liver that was clinically indistinguishable from metachronous CRLM. The patient underwent initial surgery for locally advanced sigmoid colon cancer (T4bN0M0), and during the 1-year follow-up, a hepatic tumor was detected in S7 on a contrast CT scan with EOB-MRI and PET/CT revealing a slightly low-intensity lesion on T1-weighted images and a high-intensity signal on T2-weighted images along with FDG accumulation in the liver nodule at S7. Based on these findings, the diagnosis of CRLM was initially made. However, microscopic examination of the tumor revealed clusters of lymphocytes without atypia consistent with RLH.

As of now, 84 reports detailing clinical features of liver RLH have been documented [3, 4]. Median age was 57 (range, 49–66) with predominant female proportion (*n* = 78, 92.8%). The etiology of RLH has been linked to various chronic inflammatory and immunological conditions, such as viral hepatitis, as well as a history of malignancy [3, 6–8]. Considering these factors, a potential etiology for this patient could be a local inflammatory process due to sigmoid colon cancer, although the detailed mechanism is unknown. Another proposed mechanism involves the influence of sex hormones, given the predominance of hepatic RLH in females [9]. Among the previous reports on RLH in the liver, only six cases including ours have discussed the differential diagnosis between colorectal cancer and liver metastasis (Table 1) [7, 10–12]. Perhaps not surprisingly, the majority of the patients were older adults (median age: 76 [range, 64–77]) compared to all reported RLH cases, and all of them were female. More importantly, earlier studies indicated that radiological findings of RLH closely resembled those of CRLM, posing a challenge in the differential diagnosis. Specifically, imaging studies have shown hypoechoic mass on US and a low-density lesion on CT with mild to moderate enhancement, as well as a 10–20 mm nonspecific lesion on MRI, that is, hypointense on T1-weighted images and hyperintense on T2-weighted images [11]. Given that physician’s concern of recurrence, particularly in cases of highly advanced tumors with a short interval since the initial surgery like our case, it is clinically justifiable to classify a suspicious lesion indicating metastasis as such when observed on imaging.

**Table 1:** Previous four cases of RLH mimicking CRLM.

Author	Publish year	Age	Sex	Primary tumor	Contrast CT	T1 image MRI	T2 image MRI	PET-CT	Location of RLH	RLH size	Timing	Interval from initial surgery
Sato *et al*. [7]	2006	75	Female	NA	NA	NA	NA	NA	NA	14 mm, 20 mm	Synchronous	NA
Takahashi *et al*. [10]	2006	77	Female	Ascending, stage unknown	Low density	Slightly hypotense	Hyperintense	NA	S3	15 mm	Synchronous	NA
		64	Female	Ascending, pT2	Low density	Slightly hypotense	Hyperintense	NA	S1	9 mm	Metachronous	10 years
Kobayashi *et al*. [11]	2011	68	Female	NA	Low density	Slightly hypotense	Hyperintense	NA	S5	15 mm	Metachronous	NA
Cambruzzi *et al*. [12]	2022	78	Female	NA, pT1	NA	Hypointense	Hyperintense	NA	S8	16 mm	Metachronous	12 months
Our case		78	Female	Sigmoid, pT4	Low density	Hypointense	Hyperintense	SUV 3.91	S7	10 mm	Metachronous	12 months

NA: not assessed or mentioned;

Generally, partial hepatectomy is considered a suitable approach for treating CRLM or other benign liver tumors [13]. Nonetheless, we opted to perform a posterior sectionectomy in our patient. The rationale for choosing posterior sectionectomy was attributed to the tumor being located adjacent to the RHV and closer to the hepatic hilum. The postoperative course was uneventful, and the patient was discharged on the 11th postoperative day without experiencing any complications. This outcome aligns with that of a previous report that supported the favorable outcomes associated with the Glissonian pedicle approach for central tumors and those deeply located in the right liver [14]. As such, sectionectomy using Glissonian approach has been shown to be a technique used to improve perioperative outcomes in liver tumors located in anatomically challenging areas.

## Conclusion

RLH of the liver is clinically indistinguishable from CRLM. As such, RLH after colorectal cancer resection is an unsolved clinical challenge, although RLH of the liver discovered during follow-up after the initial surgery for advanced colon cancer is rare.

## Data Availability

The data will be available upon reasonable request to the corresponding author.
